# Responses to Maximal Strength Training in Different Age and Gender Groups

**DOI:** 10.3389/fphys.2021.636972

**Published:** 2021-02-17

**Authors:** Hans Torvild Kittilsen, Sannija Goleva-Fjellet, Baard Ingegerdsson Freberg, Iver Nicolaisen, Eva Maria Støa, Solfrid Bratland-Sanda, Jan Helgerud, Eivind Wang, Mona Sæbø, Øyvind Støren

**Affiliations:** ^1^Department of Sport and Outdoor Life Studies, University of South-Eastern Norway, Bø, Norway; ^2^Department of Natural Sciences and Environmental Health, University of South-Eastern Norway, Bø, Norway; ^3^The Norwegian Biathlon Association, Oslo, Norway; ^4^Top Sports Medical Office, Tønsberg, Norway; ^5^Department of Circulation and Medical Imaging, Faculty of Medicine Trondheim, Norwegian University of Science and Technology, Trondheim, Norway; ^6^Myworkout, Medical Rehabilitation Centre, Trondheim, Norway; ^7^Faculty of Health and Social Sciences, Molde University College, Molde, Norway; ^8^Division of Geriatrics, Department of Internal Medicine, University of Utah, Salt Lake City, UT, United States

**Keywords:** training adaptations, baseline strength, leg-press, gender, aging, gene polymorphisms

## Abstract

**Purpose:**

The present study aimed to investigate the potential impact of age, gender, baseline strength, and selected candidate polymorphisms on maximal strength training (MST) adaptations.

**Methods:**

A total of 49 subjects (22 men and 27 women) aged 20–76 years, divided into five age groups, completed an 8 weeks MST intervention. Each MST session consisted of 4 sets with 4 repetitions at ∼85–90% of one-repetition maximum (1RM) intensity in leg-press, three times per week. 1RM was tested pre and post the intervention and blood samples were drawn to genotype candidate polymorphisms *ACE* I/D (rs1799752), *ACTN3* R577X (rs1815739), and *PPARGC1A* Gly482Ser (rs8192678).

**Results:**

All age groups increased leg-press 1RM (*p* < 0.01), with a mean improvement of 24.2 ± 14.0%. There were no differences in improvements between the five age groups or between male and female participants, and there were no non-responders. Baseline strength status did not correlate with 1RM improvements. *PPARGC1A* rs8192678 T allele carriers had a 15% higher age- and gender corrected baseline 1RM than the CC genotype (*p* < 0.05). C allele carriers improved 1RM (%) by 34.2% more than homozygotes for the T allele (*p* < 0.05).

**Conclusion:**

To the best of our knowledge, this is the first study to report improvement in leg-press maximal strength regardless of gender, baseline strength status in all age groups. The present study is also first to demonstrate an association between the *PPARGC1A* rs8192678 and maximal strength and its trainability in a moderately trained cohort. MST may be beneficial for good health and performance of all healthy individuals.

## Introduction

Muscle strength is important for everyday functionality in all age groups. From the age of approximately 40, maximal muscle strength decreases steadily (Lindle et al., 1997; Lambert and Evans, 2002; Petrella et al., 2005; Distefano and Goodpaster, 2018), and the decrease seems to accelerate from the age of 50 to 70 (Unhjem et al., 2016). As a consequence of this, elevated risk of physical frailty, reduction in general motor function, decline in functional movement, poor balance, falls, risk of fracture, and decline in quality of life has been reported (Fiatarone et al., 1994; Kirkendall and Garrett, 1998; Kanegusuku et al., 2015; Unhjem et al., 2016). Strength training has been recommended to delay or reverse the structural and functional changes that occur with aging in the neuro-muscular system (Hakkinen et al., 2000; Petrella et al., 2005; Unhjem et al., 2016). Maximal strength training (MST) above 85% of one-repetition maximum (1RM) has been suggested to be more effective than low-intensity training regimens to improve muscle strength in both young and old (Heggelund et al., 2013). In previous strength training interventions, the effect of age on strength training adaptions has been studied in young versus old (Unhjem et al., 2015, 2016; Wang et al., 2017) or middle-aged versus old (Hakkinen et al., 2000; Sillanpaa et al., 2009), but not in a large cohort ranging from young via middle-aged to old, with the same initial training status, typical for what is observed in the population. A study showed that age affects changes in 1RM with young subjects having a greater increase in 1RM compared with older subjects (Lemmer et al., 2000). Some MST studies have included both males and females; however, these studies have not reported any difference between genders (Lemmer et al., 2000; Storen et al., 2008; Sunde et al., 2010). A large inter-individual variability has been observed in different muscle strength-related phenotypes in response to the same strength training (Hubal et al., 2005, 2011). Heritability estimates for general muscle strength have been reported to range from 30% to 60% (Perusse et al., 1987), and overall heritability of strength-related phenotypes has been estimated to be around 50% (Zempo et al., 2017). However, heritability impact on responses to strength training seems to depend highly on the measured phenotypes (Arden and Spector, 1997; Thomis et al., 1998a,b; Calvo et al., 2002). The genetic component in muscle strength-related phenotypes seems thus to be strong, but not fully understood (Roth, 2012). Currently, more than 200 polymorphisms have been associated with strength/power phenotypes (Moreland et al., 2020), especially in relation to athletic performance (Ahmetov and Fedotovskaya, 2015; Maciejewska-Skrendo et al., 2019). Some of the most extensively studied polymorphisms in association with various aspects of exercise genetics are *ACE* I/D (rs1799752), *ACTN3* R577X (rs1815739), and *PPARGC1A* Gly482Ser (rs8192678) (Ahmetov and Fedotovskaya, 2015). These polymorphisms were selected based on previous research showing potential associations with baseline muscle strength (Erskine et al., 2014; Wilson et al., 2019) and/or strength trainability (Pickering and Kiely, 2017; Seto et al., 2019; Alvarez-Romero et al., 2020), and/or strength/power athlete status (Gineviciene et al., 2016; Tharabenjasin et al., 2019b; Wilson et al., 2019; Moreland et al., 2020). Furthermore, previous studies investigating *ACE* I/D and *ACTN3* R577X polymorphisms have produced varying results (Clarkson et al., 2005; Pescatello et al., 2019; Romero-Blanco et al., 2020). As to the *PPARGC1A* rs8192678, although associations with strength/power traits have been found, these are mostly related to the strength/power athlete status (Tharabenjasin et al., 2019a; Moreland et al., 2020). Few, if any, have investigated the effects of this polymorphism on muscle strength in a healthy adult population.

*ACE* gene codes for the angiotensin-converting enzyme involved in the regulation of blood pressure (Coates, 2003) and exhibiting a local effect on skeletal muscle function (Jones and Woods, 2003). The insertion/deletion (I/D) polymorphism (rs1799752) within the *ACE* gene has been widely investigated in relation to various skeletal muscle phenotypes (Ahmetov et al., 2013; Pereira et al., 2013b; Erskine et al., 2014; Wagle et al., 2018). *ACTN3*, coding for α-actinin-3 protein, has been described as “gene for speed” (North et al., 1999; MacArthur and North, 2004). R577X (rs1815739), a single nucleotide polymorphism (SNP) leading to a premature stop codon, has been associated with various muscle phenotypes in athletes (Yang et al., 2009; Eynon et al., 2013) as well as in the general population (Ahmetov et al., 2013; Pereira et al., 2013b; Del Coso et al., 2018; Houweling et al., 2018; Pickering and Kiely, 2018). Around 18% of the population of European ancestry are homozygous for the 577X allele, thus lacking the α-actinin-3 (Kikuchi and Nakazato, 2015; ENSMBL, 2020). This affects several aspects of muscle metabolism, leading to lower muscle strength and mass, among others (Seto et al., 2019). *PPARGC1A* gene codes for the peroxisome proliferator-activated receptor-gamma coactivator-1α (PGC-1α), enriched in metabolically active tissues (Liang and Ward, 2006). PGC-1α have a range of functions, including being a master regulator of mitochondrial biogenesis (Di Meo et al., 2017). Several PGC-1α isoforms exist, exhibiting actions through different pathways (Martinez-Redondo et al., 2016). Although most of the findings relate to adaptations to aerobic exercise (Lira et al., 2010; Steinbacher et al., 2015) and athletic ability (Tharabenjasin et al., 2019a), PGC-1α may also mediate the adaptations to resistance training (Ruas et al., 2012; Gineviciene et al., 2016). A common coding SNP within the *PPARGC1A* gene is the rs8192678 polymorphism (Nitz et al., 2007), more known as Gly482Ser, a missense mutation where serine (Ser) substitutes glycine [Gly; NCBI (2018)]. Despite receiving a lot of scientific attention (Kim et al., 2014; Ben-Zaken et al., 2015, 2019; Gineviciene et al., 2016; Ginszt et al., 2018; Fluck et al., 2019), it is still uncertain how candidate genetic variants may influence MST responses in a cohort with a training status typical for their age.

Previously, MST has been shown to effectively improve maximal muscle strength in different cohorts ranging from patients to athletes, and from young to old of both genders (Storen et al., 2008; Sunde et al., 2010; Helgerud et al., 2011; Barrett-O’Keefe et al., 2012; Wang et al., 2017). The effect of MST is thus relatively well documented and so are the possible differences in training adaptions in young versus old. However, no previous studies have systematically investigated the adaptions to MST and in different age groups from young adults to old in 10 years- steps in the same study. Furthermore, no previous studies have also tested gender differences and the impact of selected genetic variants in the same study. This combination is therefore the main novelty of the present study. This may be important for the health and functional benefits an individual can expect. Thus, the purpose of the present study was to investigate the effect of age, gender, baseline strength status, and candidate gene status on the adaptability to leg-press MST. Specifically, we hypothesized that (1) MST would lead to an increase in maximal strength in all age cohorts; (2) the increase in maximal strength would not be different between males and females; (3) MST would improve maximal strength more in young (20–29 and 30–39) than in middle-aged (40–49 and 50–59) or old (60+); (4) the improvements in maximal strength after MST would not be affected by the selected key genetic variants.

## Materials and Methods

### Subjects

A total of 76 healthy subjects (33 men and 43 women) with age ranging from 20 to 76 years, were included in the present study. Most of the subjects were active and familiar with some sort of strength training, but none of the subjects had experience with MST in leg-press over six or more months before the study. They continued with their normal activities and diet during the study period, no supplementations were provided or recommended. Of these, twenty-seven subjects (11 males and 16 females) aged 21 to 67 years did not complete the study. From the 27 drop-outs, 18 did not meet the inclusion criteria for adherence due to common sickness, lack of time or motivation, or other known reasons not related to the intervention itself. Nine dropouts were directly related to the intervention. Subjects characteristics that completed the MST intervention are presented in Tables 2, 3. Subjects that completed the study were divided into five age groups with 10 years age-span in each, except the oldest group ranging from 60–76 years. Each age group was matched for baseline 1RM in leg-press, corrected for age, gender (Table 1) and body mass (Table 2). The correction was based on previously reported values in males and females with different age [Lindle et al. (1997), Hakkinen et al. (2000), Petrella et al. (2005), Reynolds et al. (2006), Reid et al. (2008), Dey et al. (2009), Unhjem et al. (2016), and Wang et al. (2017)], and mean age and gender differences were calculated based the results and the number of participants in these previous studies.

**TABLE 1:** Age and gender correction table for maximal strength in leg-press.

Age group	20–29	30–39	40–49	50–59	60–70+
**Gender**					
Male	1	1	1	1	1
Female	0.6	0.6	0.6	0.6	0.6
**Age**	1	0.98	0.92	0.88	0.82

*Values are based on means of the results i.e., gender differences, adjusted for the number of participants, from the following studies: Lindle et al. (1997), Hakkinen et al. (2000), Petrella et al. (2005), Reynolds et al. (2006), Reid et al. (2008), Dey et al. (2009), Unhjem et al. (2016), and Wang et al. (2017).*

**TABLE 2:** Age, body weight (BW) at baseline, and maximal strength (1RM) in leg-press at pre- and post-tests, and percentage improvements (Δ1RM) in maximal strength.

Age group:	1 (*n* = 10)	2 (*n* = 9)	3 (*n* = 12)	4 (*n* = 8)	5 (*n* = 10)	Total (*n* = 49)
**Age (yrs)**	25.62.8	33.92.8	44.23.2	53.53.0	70.34.3	45.316.0
**BW (kg)**	74.48.9	83.511.8	74.412.5	80.815.1	70.34.2	76.312.2
**Pre-1RM (kg)**	224.553.3^#^	362.2135.3	255.886.9	240.677.6	191.050.8^#^	253.399.6
**1RMcorr (kg)**	18.14.7	20.84.9	20.54.3	18.33.4	19.55.5	19.54.6
**Post-1RM (kg)**	267.059.1*	443.9136.7*	333.13115.2*	290.6399.6*	231.553.2*	313.3118.4*
**Δ1RM (%)**	19.57.4	25.515.0	30.919.2	20.29.3	22.8613.3	24.214.0

Data are presented as mean ± SD, standard deviation. BW, body weight; Yrs, years; Kg, kilograms; 1RM, one-repetition maximum; corr., baseline 1RM corrected for age, gender, and body weight raised to the power of 0.67. *p < 0.01different from pre-test. ^#^*p* < 0.01different from group 2 at baseline.

**TABLE 3:** Age, body weight (BW) at baseline, the percentage change in BW, and leg-press maximal strength (1RM) and percentage improvements (Δ1RM) in maximal strength by gender (*N* = 49).

	Age (yrs)	BW (kg)pre	Δ BW (%)	1RM (kg)pre	Δ 1RM (%)
**Males (*N* = 22)**	43.313.8	83.111.8	1.13.0	315.2112.6	26.215.3
**Females (*N* = 27)**	47.017.7	70.79.5*	−0.32.3*	202.846.4*	22.613.0

Results are mean ± SD, standard deviation, and percent change from pre to post-intervention. Yrs, years; BW, body weight; 1RM, one-repetition maximum in leg-press; Kg, kilograms; % percent. *P < 0.01 different from males.

Inclusion criteria were general good health status with no contra-indications for MST and testing, assessed by the study‘s physician, and compliance of at least 80% of all training sessions. Exclusion criteria included any injury or illness that could prevent subjects from performing in MST or testing in leg-press or compliance of less than 80% of all training sessions. Informed consent was obtained from all subjects, and the study was approved by the institutional review board of Telemark University College (now the University of South-Eastern Norway) and the Norwegian Centre for Research Data (NSD, reg 45185/3/AH). The study was also registered in Clinical trials (NCT02589990).

### Study Timeline

The subjects performed pre-testing 2–4 days before the 8-week MST intervention, and post-tests 2–5 days after the last training session. Subjects were instructed not to exercise the last 24 h before the test days, not to eat within 2–4 h before the tests, and only to drink water for the last 2 h before the testing procedures.

### Testing

Pre- and post-tests were identical and performed at the same time of day ±1 h. A general warm-up for 10 min performed as cycling, walking or running was performed at a moderate intensity. After the general warm-up, a specific warm-up was performed in the leg-press machine (OPS161 interchangeable leg-press, Vertex United States). This included sets of 10, 5, and 3 repetitions at approximately 50, 60, and 70% of 1RM, respectively. The estimates of 1RM before the first 1 RM test were based on age, gender, body weight, and training history. There were 3 min of rest between each set. Following this, 1RM was assessed by first one repetition at approximately 80% 1RM, and then one and one repetition at weight loads increased by 5–15 kg from the previous lift, separated by 3 min rest until reaching 1RM. Each lift was performed with a controlled slow eccentric phase, a complete stop of movement for approximately 1 s in the lowest position (90 degrees between femur and tibia), followed by a maximal mobilization of force in the concentric phase, as described in Storen et al. (2008) and (Sunde et al., 2010). Lifting time and distance were measured using the Muscle lab system (Ergotest Innovation A.S., Porsgrunn, Norway) to control the work distance.

### Maximal Strength Training (MST)

The MST intervention lasted for 8 weeks and included three MST-sessions per week with at least 1 day of rest between each session. Participants were instructed to maintain their habitual training as normal, and both the MST intervention and habitual training was logged. Each session consisted of a general warm-up for 10 min at moderate intensity and then three 10-repetition warm-up sets in leg-press with increasing load (30–70% 1RM). After the warm-up, participants performed four sets of 4RM in the leg-press, with 90 degrees between femur and tibia, divided by 3 min of rest between sets. Every time a subject managed to do five repetitions during a set, 2.5–5 kg were added for the next set. Guidance and instruction were given to all subjects during the training period.

### DNA Sampling and Genotyping

Of the 76 subjects recruited, 72 consented to genetic testing. Venous blood was collected in EDTA tubes from all participants prior to the admission to the exercise intervention. The samples were stored at −20°C until the genomic DNA was extracted from 100 μl of blood using the DNeasy Blood & Tissue Kit (Qiagen, MD, United States) according to the manufacturer’s instructions.

The rs4343 polymorphism in the *ACE* gene, which might be the best proxy to *ACE* I/D polymorphism (Abdollahi et al., 2015), was analyzed to determine the I/D genotype. Genotyping for all polymorphisms was performed using TaqMan^®^ SNP Genotyping Assay. Assay ID were as follows: C__11942562_20 for *ACE* rs4343; C____590093_1 for the *ACTN3* R577X and C___1643192_20 for the *PPARGC1A* rs8192678 (Thermo Fisher Scientific, MA, United States). qPCR was carried out on the StepOnePlus^TM^ Real-Time PCR System (Applied Biosystems^®^, CA, United States), and genotype calling was performed by StepOne Software v2.0. The final reaction volume was 15 μl and contained 8.44 μl Genotyping Master Mix, 0.42 μl Assay mix (40×), 6.33 μl distilled H_2_0 and ∼100 ng of DNA template. Following cycling conditions were used: 30 s at 60°C followed by initial denaturation step for 10 min at 95°C; 40 cycles of denaturation at 95°C for 15 s followed by annealing at 60°C for 1 min in cycling stage, and a final post-read step for 30 s at 60°C.

### Statistical Analysis

Data were tested for normality by use of QQ-plot and the Kolmogorov-Smirnov test and found to be normally distributed for the main variable 1RM, corrected for age and gender (1RMcorr). A general linear model with Tukey *post hoc* analyses for age groups was used to assess 1RM and Δ1RM results. Independent t-tests were used to compare males and females overall since the sample size was too low to assess potential gender differences in each age group. Associations between the genotypes and continuous variables, and the alleles and continuous variables were analyzed by one-way ANOVA and two-tailed independent sample t-tests, respectively. Correlation analyses were performed by use of the Pearson correlation test. Pearson’s Chi-square test (χ^2^) was applied to test for the Hardy-Weinberg equilibrium (HWE) for all polymorphisms and the differences in categorical variables. The significance level was set to *p* < 0.05 in two-tailed tests. All statistical analysis were performed by the use of IBM SPSS Statistics, version 25 (Chicago, IL, United States).

In the present study, the sample size for genetic association studies is relatively small. Therefore, to determine the magnitude of differences, also Cohen’s d effect size was calculated for baseline strength (1RMcorr) and Δ1RM (%) across phenotypes. Effect sizes were interpreted as: *d* = < 0.35 (trivial), *d* = 0.35–0.80 (small); *d* = 0.80–1.50 (moderate); *d* = > 1.50 (large effect size) (Rhea, 2004).

## Results

Forty-nine subjects (22 males and 27 females) aged 20 to 76 years (45.3 ± 16.0) completed the 8-week three times per week MST intervention. There was no difference in baseline characteristics between completers and non-completers.

Baseline 1RM in absolute values (kg) decreased with increasing age (*p* < 0.01) from group 2 (33.9 ± 2.8 years). Baseline 1RM corrected for age, gender and body mass scaled to the power of 0.67 (kg kg^–0^.^67^) was not significantly different between any of the age groups (Table 2).

**FIGURE 1:**
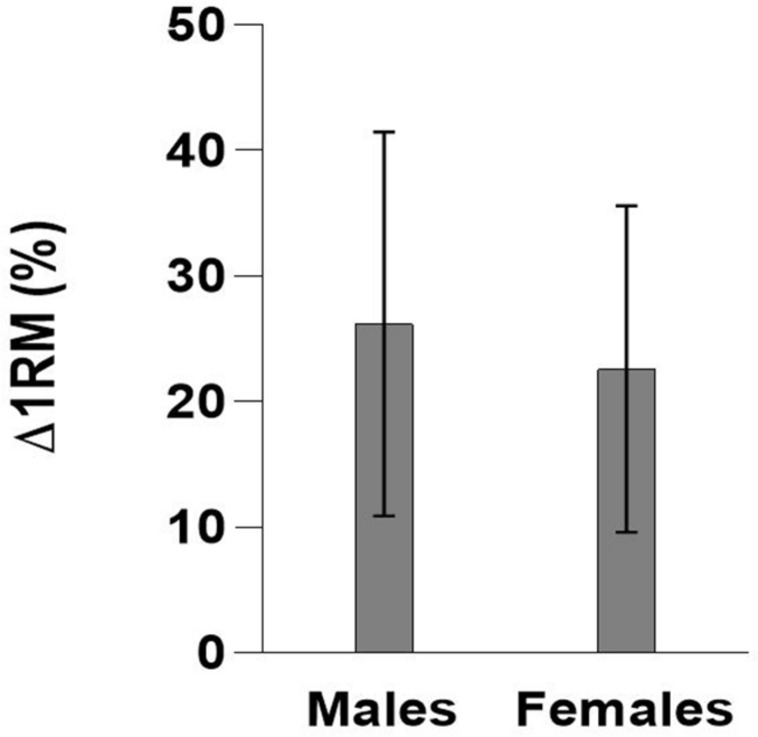
Mean improvements (%) in one-repetition maximum (Δ1RM) ± standard deviation (SD) following an 8-week maximal strength training program by gender.

After the intervention, there was a mean improvement in 1RM leg-press of 24.2 ± 14.0% (*p* < 0.01), with no participants having less than 7% improvement. In relative terms (%), there were no significant differences in Δ1RM between any of the age groups (Table 2 and Figure 1). No changes in body mass in any of the groups were found following the intervention.

**FIGURE 2:**
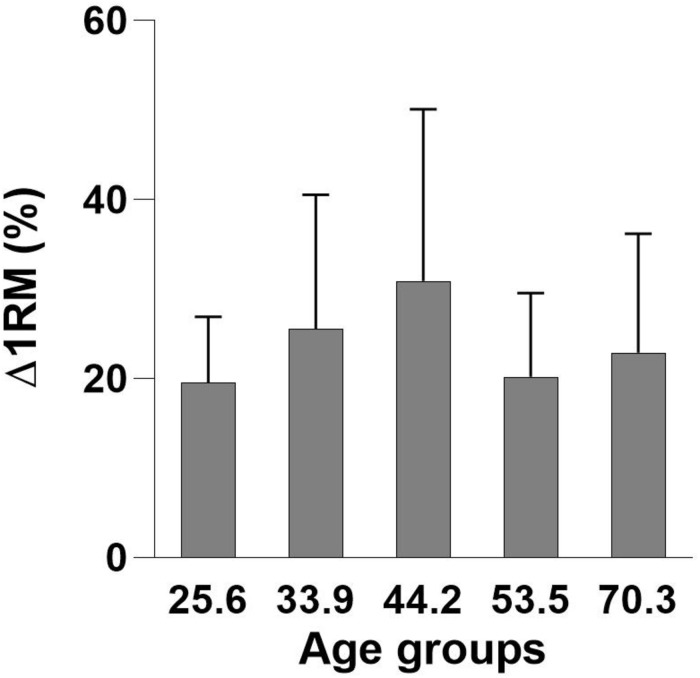
Mean age group improvements (%) in one-repetition maximum (Δ1RM) ± standard deviation (SD).

At baseline, males were heavier and had higher 1RM in absolute values (kg) than females (*p* < 0.01). Independent of age groups, males improved 1RM by 26.2% ± 15.3%, whereas the females improved 1RM by 22.6% ± 13.0% (Table 3 and Figure 2), which was not significantly different (*p* = 0.56). There was no significant correlation between baseline 1RM and relative improvement in Δ1RM (%) (*r* = 0.25, *p* = 0.08).

All three gene polymorphisms were successfully genotyped. Baseline MST and genotype data for *ACTN3* R577X (rs1815739) and *PPARGC1A* Gly482Ser (rs8192678) were available for 72 individuals, and 70 – for the *ACE I*/D (rs1799752) polymorphism. Genotype distributions for all polymorphisms among those that completed the intervention (*N* = 49), are displayed in Table 4. Minor allele frequencies for these polymorphisms were 52% for the *ACE* I allele, 48% for the *ACTN3* X allele and 40% for the *PPARGC1A* T (Ser) allele. The frequencies did not differ significantly when all subjects with the baseline genetic data were included. Also, the genotype frequencies were consistent with Hardy-Weinberg Equilibrium (*P* > 0.05).

**TABLE 4:** Genotype distributions for *ACE* I/D, *ACTN3* R577X, and *PPARGC1A* rs8192678 polymorphisms (*N* = 49).

*ACE*		*ACTN3*		*PPARGC1A*	
**DD**	25%	**RR**	27%	**CC**	41%
**ID**	45%	**RX**	51%	**CT**	39%
**II**	30%	**XX**	22%	**TT**	20%
**D al.**	48%	**R al.**	52%	**C al.**	60%
**I al.**	52%	**X al.**	48%	**T al.**	40%

*Results are displayed as percentages (%); Al.-allele.*

*PPARGC1A* Gly482Ser (rs8192678) T allele carriers demonstrated 15.0% higher baseline 1RMcorr compared to the CC genotype. Also, the participants with CT genotype were 17.9% stronger at baseline (1RMcorr) compared to the wild type CC counterparts (*p* < 0.05) (Table 5). C-allele carriers, on the contrary, showed 34.2% higher improvements in Δ1RM (%), compared to the homozygotes for the minor allele i.e., the TT genotype (*p* < 0.05) (Table 5). 1RMpre and 1RMpost values for the *ACE* I/D (rs1799752), *ACTN3* R577X (rs1815739), and *PPARGC1A* Gly482Ser (rs8192678) polymorphisms are displayed as Supplementary Figure 1.

**TABLE 5:** Associations between the *PPARGC1A* rs8192678 polymorphism and leg-press maximal strength and percentage improvements in maximal strength.

	**1RMcorr (kg)**	**Δ1RM (%)**
***PPARGC1A***		
CC	17.8 ± 4.4^*/#^	29.3 ± 17.5
CT	**21.3 ± 4.5^#^**	22.0 ± 11.7
TT	20.0 ± 4.1	**18.2 ± 4.6¤**
C allele	19.5 ± 4.7	**25.7 ± 15.2¤**
T allele	**20.7 ± 4.4^∗^**	20.7 ± 9.9

*p = 0.027. ^#^*p* = 0.042. ¤*p* = 0.011.

No significant associations were found between the *ACE* I/D (rs1799752), *ACTN3* R577X (rs1815739), and baseline 1RMcorr or Δ1RM (%). However, participants with *ACTN3* RR genotype demonstrated a non-significant 46.5% larger increase in Δ1RM on average, compared to their XX counterparts. This corresponds to a moderate effect size measured in Cohen’s d (Supplementary Table 2). A summary table of all genotype/allele combinations for the *ACE* I/D (rs1799752), *ACTN3* R577X (rs1815739) and *PPARGC1A* Gly482Ser (rs8192678) polymorphisms, and 1RMcorr and Δ1RM (%) can be found in the Supplementary Table 1.

## Discussion

The main findings of the present study were that MST-induced increases in leg-press 1RM were similar regardless of age, gender, baseline strength status or most of the selected candidate polymorphisms. The first hypothesis that all age cohort would improve in maximal strength was confirmed. The second hypothesis that an increase in maximal strength would not be different between males and females was also confirmed. The third hypothesis that young would improve more than old was rejected. The fourth hypothesis that improvement in maximal strength would not be affected by the selected polymorphisms was only partly confirmed. C allele carriers for the *PPARGC1A* Gly482Ser (rs8192678) improved 1RM more than those with the TT genotype. At baseline, T allele carriers had higher 1RMcorr compared to those with the CC genotype.

The results from the present study show the same results in maximal strength adaptations as previously found in VO_2*max*_ adaptations in Storen et al. (2017). Although middle-aged and old had lower baseline values in these two studies, the relative improvements were just as good in untrained and moderately trained at older ages. To our knowledge, this is the first study to report similar training responses in all age groups from young adults in their twenties and thirties via middle-aged in their forties and fifties and up to older adults in their sixties and seventies.

That MST was an effective method to improve maximal strength was in the present study shown by no non-responders to the MST program, with the smallest improvement being 7.4%. Furthermore, the improvements in maximal strength were rather homogenous, with a coefficient of variance of 8.7%.

### The Impact of Age, Gender, and Selected Candidate Polymorphisms on Baseline 1RM

As expected, 1RM (kg) decreased with advancing age at baseline (Table 2). The 1.3% decrease per year in the current study from young (33.9 years) to old (70.3 years) is in line with previous studies (Lindle et al., 1997; Lambert and Evans, 2002; Petrella et al., 2005; Distefano and Goodpaster, 2018), but the decrease in the present study is evenly distributed among age groups. The results show a 1.2% decrease from 53.5 years to 70.3 years of age, while some studies also show an accelerated drop in muscle strength from 50 to 70 years (Lindle et al., 1997; Lambert and Evans, 2002; Petrella et al., 2005; Distefano and Goodpaster, 2018). In the present study, males were 56% stronger than females, expressed in absolute values (kg). This corresponds well with the findings of Petrella et al. (2005) and Reynolds et al. (2006), showing approximately 50–65% higher 1RM in lower extremities in males than in females.

Individuals with *PPARGC1A* Gly482Ser (rs8192678) CC genotype had lower 1RMcorr at baseline compared to both CT genotype counterparts and T-allele carriers. These associations remained significant when the data for all participants with the genetic data for the rs8192678 available were analyzed (*N* = 72). This may indicate that the Ser-encoding T allele might be favorable for muscle strength not only in power/strength athletes but also in the general population. Interestingly, when comparing the genotype frequencies for the rs8192678 between the present cohort and a highly trained Scandinavian cross-country athlete cohort (Johansen et al., 2020), significant differences were found. Although in both cohorts the minor T allele frequency was comparable (∼39%), the TT genotype was underrepresented among the endurance athletes compared to the cohort investigated in the present study representing the general public (3% vs. 20%, respectively). These findings are in line with previous studies (Ahmetov and Fedotovskaya, 2015). The rs8192678 polymorphism has been associated with differences in *PPARGC1A* mRNA expression, with lower expression among carriers of T allele (Vandenbeek et al., 2018). Gene expression responses may be important for muscle adaptations in response to different modes of exercise (Silvennoinen et al., 2015).

### Improvements in 1RM

The average relative improvements in 1RM by ∼24%, was not different between the age groups after 8 weeks of MST. The size of the average improvement is in line with comparable studies on MST, showing improvements in the range of 23–33% (Storen et al., 2008; Sunde et al., 2010; Barrett-O’Keefe et al., 2012).

To our knowledge, this is the first study to report similar training responses in all age groups from young adults in their twenties and thirties via middle-aged in their forties and fifties and up to older adults in their sixties and seventies. The oldest group improved 1RM to the same extent as the mean of the other four age groups. The present results are in line with some studies comparing young and old, like Hakkinen et al. (2000), but differ from Lemmer et al. (2000) and Petrella et al. (2005) showing better adaptations in young than old.

That males and females improved relative 1RM to the same extent was as expected, and in line with previous studies. No gender differences in Δ1RM% were found in Hakkinen et al. (2000), Storen et al. (2008), Sunde et al. (2010), Kanegusuku et al. (2015), Berg et al. (2018), and Winther et al. (2018).

When corrected for age, gender and body mass (1RMcorr), baseline 1RM indicates the participants’ baseline strength status. In light of this, it was somewhat surprising that initial baseline strength status did not significantly affect 1RM improvements. In a previous study on VO_2*max*_ adaptations to endurance training in different age groups (Storen et al., 2017), initial training status was found to significantly affect training adaptations. This should also be expected in MST interventions, as untrained and trained in previous studies have shown rapid improvements in neural adaptations during the first 2–4 weeks of this type of training (Hakkinen et al., 2000; Storen et al., 2008; Unhjem et al., 2015, 2016; Wang et al., 2017).

Bodyweight did not change in the present study, and this may support the assumption that it is predominately the neural adaptions and changes in recruitment patterns, which have led to increased 1RM. However, any change in body composition cannot be excluded in the present study and this is in line several other studies (Lemmer et al., 2000; Sunde et al., 2010; Barrett-O’Keefe et al., 2012; Unhjem et al., 2016).

Carriers of the *PPARGC1A* Gly482Ser (rs8192678) T allele had higher baseline 1RMcorr compared to the CC genotype. The T allele, more widely known as the Ser allele, has been associated with strength/power athlete status (Gineviciene et al., 2016; Moreland et al., 2020), indicating an advantageous effect on muscle strength not only in athletes but also among the general public. On the other hand, C allele carriers, possessing lower 1RMcorr at baseline, demonstrated larger improvements in 1RM compared to the TT genotype in the present study. These differences could theoretically be attributable to a larger potential for improvements, due to lower muscle strength at baseline in C allele carriers. However, baseline 1RM and improvements in 1RM did not correlate in the present study. Resistance training has been shown to induce expression of an isoform of the protein coded by the *PPARGC1A* gene (i.e., PGC-1α4) that regulates muscle hypertrophy (Ruas et al., 2012). The polymorphism is known to influence mRNA expression (Vandenbeek et al., 2018). However, to the best of authors’ knowledge, it is not known whether the Gly482Ser polymorphism may influence the expression of the hypertrophy-specific isoform.

No significant associations between *ACE* I/D (rs1799752) and *ACTN3* R577X (rs1815739) polymorphisms and baseline or Δ1RM were found in the present study. Of these, especially the *ACTN3* R577X (rs1815739) polymorphism has been shown to have a range of effects on various muscle phenotypes, such as improvements in strength or muscle function (Pickering and Kiely, 2017, 2018; Del Coso et al., 2018). Previous studies indicate that the R allele may be advantageous for an increased maximal dynamic strength (Delmonico et al., 2007; Pereira et al., 2013a; Weyerstrass et al., 2018). The X allele, on the other hand, appears to be detrimental for strength/power athlete status (Roth et al., 2008) and other aspects of muscle strength (Pickering and Kiely, 2018). In the present study, Cohen’s *d* for differences in Δ1RM between the RR and XX genotypes indicates a moderate negative effect for the latter group, as presented in Supplementary Tables 1, 2, which points toward improved response to resistance training among the carriers of the R allele.

### Strengths and Limitations

Of the 76 participants initially recruited, 49 subjects completed the study and 27 did not. From the 27 drop-outs, 18 did not meet the inclusion criteria for adherence due to reasons not related to the intervention itself, while nine individuals – due to reasons related to the intervention. It was chosen to rather take subjects out to early from the study than to risk any injury, and this had an impact on the nine study-related drop-outs. It is important to note that the drop-out rates did not differ between the age groups and appears to be in line with previous studies MST (Wang et al., 2017) and conventional strength training (Raymond et al., 2013). We thus propose that MST 2–3 times/week can still be recommended to improve muscle strength and to potentially delay age-associated decline in muscle function.

Only three, out of more than 200 genetic variants associated with strength/power phenotypes (Moreland et al., 2020), were included in the present study. *ACE* I/D (rs1799752), *ACTN3* R577X (rs1815739), and *PPARGC1A* Gly482Ser (rs8192678) were selected based on the fact, that they have been extensively investigated in the field of exercise genomics, and, have been associated with various strength-related phenotypes. To date, there are few, if any, studies investigating the effects of *PPARGC1A* Gly482Ser (rs8192678) on maximal strength in healthy adults. Furthermore, authors had previously genotyped *ACE* I/D (rs1799752), *ACTN3* R577X (rs1815739) in a large homogenous Scandinavian cohort representing the general public from the same geographic area (Goleva-Fjellet et al., 2020). Genotype frequencies of the *ACTN3* and the *ACE* polymorphisms in both studies were similar indicating that the participants in the present study were representative for the population in this region, regarding these polymorphisms. The present study is based on a sample size that is typical for a training intervention study. However, in terms of candidate gene studies, the sample size is small. A low number of participants in genetic association studies investigating complex traits tend to be vulnerable to type II error (Hong and Park, 2012). Therefore, the effect size of these relationships was also reported in the present study. The indications of greater response to resistance training in R allele carriers are thus in line with the overall impression from studies on resistance training (Seto et al., 2019). To compensate for the relatively low sample size, an ethnically homogenous study cohort was investigated.

The presented study has several limitations. The sample size is small in terms of genetic studies, and only three out of many genetic variants associated with strength/power phenotypes were included in the present study. However, the present study has also several strengths. To the best of the authors’ knowledge, this is the first study to report improvements in leg-press maximal strength regardless of gender, baseline strength, and age. No previous studies have systematically investigated the adaptions to MST and in different age groups from young adults to old in 10 years- steps in the same study, including participants 20 to 70+ years of age. Furthermore, no previous studies have also tested gender differences and the impact of selected genetic variants in the same study. This combination is the main novelty of the present study.

### Practical Implications

The present results demonstrated that MST is effective in improving maximal strength in most healthy people capable of performing MST. There were no differences in drop out between the age groups, and the dropout rate may be considered to be in line with previous MST studies such as (Wang et al., 2017). Improved muscle strength has been shown to better general motor function, maintain or increase functional movement, balance, independence, and quality of life (Fiatarone et al., 1994; Kirkendall and Garrett, 1998; Kanegusuku et al., 2015; Unhjem et al., 2016), especially among old. We, therefore, recommend MST 2–3 times per week in leg-press, squats or deadlift at all ages to delay the age-related decline in muscle strength and health. However, cautions should be taken as some may experience muscle or joint pain from this kind of exercise.

### Conclusion

To our knowledge, this is the first study to report improvements in leg-press maximal strength regardless of gender, baseline strength, and age. Improvements in MST were found in all age groups from young adults in their twenties and thirties via middle-aged in their forties and fifties and up to older adults in their sixties and seventies. Of the investigated candidate polymorphisms, only *PPARGC1A* Gly482Ser (rs8192678) demonstrated a significant effect on the baseline maximal strength and its trainability in a moderately trained cohort. This is the first study to demonstrate this association in such a cohort. Yet, the effect of this single genetic variant is likely minimal. These findings imply that most healthy individuals have great potential for maximal strength improvements and that MST may be used as a strategy for healthy aging.

## Data Availability Statement

The datasets presented in this article are not readily available due to the Norwegian Legislation regarding the publication of genetic data. Requests to access the datasets should be directed to the corresponding author.

## Author Contributions

HK, SG-F, ØS, MS, IN, and EW participated significantly in the planning and designing of the study, as well as the data analyzing and the writing of the manuscript. HK, SG-F, ØS, MS, IN, ES, SB-S, and BF participated in the data collection. JH, ES, SB-S, and BF participated in the writing of the manuscript. All authors read and approved the manuscript.

## Conflict of Interest

The authors declare that the research was conducted in the absence of any commercial or financial relationships that could be construed as a potential conflict of interest.
