# Supplementary material for: Responses to Maximal Strength Training in Different Age and Gender Groups
Source: Front Physiol. 2021 Feb 17;12:636972. doi: 10.3389/fphys.2021.636972 (PMC7925619; doi:10.3389/fphys.2021.636972)
Supplement: Supplementary file 1 [file Table_1.DOCX]

***Figure S1*** *One-repetition maximum at baseline (1RMpre) and after (1RMpost) an 8-week maximal strength training program expressed in kilograms (kg) by genotype and alleles (al.) for PPARGC1A rs8192678, ACTN3 R577X and ACE I/D polymorphisms.*
