# Supplementary material for: Responses to Maximal Strength Training in Different Age and Gender Groups
Source: Front Physiol. 2021 Feb 17;12:636972. doi: 10.3389/fphys.2021.636972 (PMC7925619; doi:10.3389/fphys.2021.636972)
Supplement: Supplementary file 2 [file Table_2.DOCX]

**Table S1 Pairwise genotype and allele comparisons for the *ACTN3* R577X, *PPARGC1A* rs8192678 and *ACE* I/D polymorphisms, 1RMcorr (baseline) and ∆1RM (%).**

| **Pair comparisons** | **N** | **1RMcorr (baseline)** | | | | | |  | **∆1RM %** | | | | | | |
| --- | --- | --- | --- | --- | --- | --- | --- | --- | --- | --- | --- | --- | --- | --- | --- |
|  |  | **Means** | **SD** | **Diff.** | **% diff.** | **Cohen's d** | **P-value** |  | **Means** | **SD** | **Diff.** | **% diff.** | **Cohen's d** | **P-value** | |
| ***ACTN3*** |  |  |  |  |  |  |  |  |  |  |  |  |  |  | |
| RR/RX | 13/25 | 18.01/20.23 | 3.71/4.90 | 2.22 | 11.61 | 0.50 | 0.336 |  | 30.00/23.59 | 18.17/13.36 | 6.41 | 23.92 | 0.43 | 0.368 | |
| RR/XX | 13/11 | 18.01/19.52 | 3.71/4.60 | 1.50 | 8.04 | 0.37 | 0.700 |  | 30.00/18.68 | 18.17/6.66 | 11.33 | 46.54 | **0.82** | 0.121 | |
| RX/XX | 25/11 | 20.23/19.52 | 4.90/4.60 | 0.71 | 3.58 | 0.15 | 0.902 |  | 23.59/18.68 | 13.36/6.66 | 4.92 | 23.27 | 0.42 | 0.587 | |
| R al./XX | 38/11 | 19.47/19.52 | 4.60/4.60 | 0.05 | 0.25 | 0.01 | 0.976 |  | 25.79/18.68 | 15.24/6.66 | 7.11 | 31.99 | 0.52 | 0.141 | |
| RR/X al. | 13/36 | 18.01/20.01 | 3.71/4.76 | 2.00 | 10.53 | 0.45 | 0.177 |  | 30.00/22.09 | 18.17/11.85 | 7.91 | 30.38 | 0.58 | 0.082 | |
| ***PPARGC1A*** |  |  |  |  |  |  |  |  |  |  |  |  |  |  | |
| CC/CT | 20/19 | 17.77/21.26 | 4.37/4.49 | 3.49 | 17.89 | **0.80** | 0.042* |  | 29.26/22.00 | 17.54/11.73 | 7.25 | 28.30 | 0.49 | 0.230 | |
| CC/TT | 20/10 | 17.77/19.53 | 4.37/4.11 | 1.76 | 9.46 | 0.42 | 0.554 |  | 29.26/18.21 | 17.54/4.62 | 11.05 | 46.55 | 0.77 | 0.102 | |
| CT/TT | 19/10 | 21.26/19.53 | 4.49/4.11 | 1.73 | 8.47 | 0.40 | 0.573 |  | 22.00/18.21 | 11.73/4.62 | 3.80 | 18.87 | 0.39 | 0.756 | |
| C al./TT | 39/10 | 19.47/19.53 | 4.71/4.11 | 0.06 | 0.33 | 0.01 | 0.969 |  | 25.72/18.21 | 15.25/4.62 | 7.51 | 34.21 | 0.55 | 0.011* | |
| CC/T al. | 20/29 | 17.77/20.66 | 4.37/4.37 | 2.90 | 15.07 | 0.67 | 0.027* |  | 29.26/20.70 | 17.54/9.93 | 8.56 | 34.28 | 0.67 | 0.058 | |
| ***ACE*** |  |  |  |  |  |  |  |  |  |  |  |  |  |  | |
| DD/ID | 12/21 | 19.47/19.98 | 5.20/3.64 | 0.51 | 2.60 | 0.12 | 0.948 |  | 21.54/23.74 | 11.15/11.50 | 2.20 | 9.74 | 0.20 | 0.904 | |
| DD/II | 12/14 | 19.47/18.91 | 5.20/5.20 | 0.56 | 2.91 | 0.11 | 0.948 |  | 21.54/28.49 | 11.15/19.32 | 6.96 | 27.81 | 0.44 | 0.433 | |
| ID/II | 21/14 | 19.98/18.91 | 3.64/5.20 | 1.07 | 5.50 | 0.25 | 0.776 |  | 23.74/28.49 | 11.50/19.32 | 4.75 | 18.19 | 0.32 | 0.599 | |
| D al./II | 31/16 | 19.83/18.97 | 4.23/5.02 | 0.86 | 4.41 | 0.19 | 0.54 |  | 23.22/27.26 | 11.55/18.33 | 4.04 | 15.99 | 0.29 | 0.36 | |
| DD/I al. | 12/35 | 19.47/19.56 | 5.20/4.29 | 0.09 | 0.43 | 0.02 | 0.956 |  | 21.54/25.64 | 11.15/15.04 | 4.11 | 17.40 | 0.29 | 0.392 | |
| Corr- corrected for age, gender and body weight raised to the power of 0.67; N- number of subjects; SD- standard deviation; Diff.- difference; al.- allele; P-values are corrected for multiple testing where appropriate (Tukey); Cohen’s d effect size: d= <0.35 (trivial), d= 0.35-0.80 (small); d= 0.80-1.50 (moderate; as defined by Rhea (2004) specifically for strength training); * P<0.05 | | | | | | | | | | | | | | |  |
